# Development and validation of the scale for symptom clusters in patients with myasthenia gravis

**DOI:** 10.1186/s12883-023-03240-4

**Published:** 2023-05-19

**Authors:** Fan Shen, Lu-Hong Hu, Hai-Shan Huang, Ling Li

**Affiliations:** 1 Nursing Department, Tongji Hospital, Tongji Medical College, Huazhong University of Science and Technology, 1095 Jiefang Avenue, Qiaokou district, Wuhan, China; 2 Department of Neurology, Tongji Hospital, Tongji Medical College, Huazhong University of Science and Technology, Wuhan, China; 3 School of Nursing, Tongji Medical College, Huazhong University of Science and Technology, Wuhan, China

**Keywords:** Myasthenia gravis, Symptom clusters, Scale, Unpleasant symptom theory, Reliability, Validity, Nursing assessment

## Abstract

**Background:**

Patients with myasthenia gravis(MG)often experience multiple symptoms concurrently, which can have an adverse effect on their quality of life(QOL). However, a specific, systemic and reliable scale for symptom clusters in MG is lacking.

**Aims:**

To develop reliable assessment scale for symptom clusters in patients with MG.

**Design:**

A cross-sectional descriptive study.

**Methods:**

Based on the unpleasant symptom theory(TOUS), the first draft of the scale was developed through review literature, qualitative interview, and Delphi expert correspondence, the items of the scale were presented and adjusted through cognitive interviews with 12 patients. To conveniently assess the validity and reliability of the scale, a cross-sectional survey was conducted in 283 patients with MG who were recruited from Tongji Hospital of Tongji Medical College, Huazhong University of Science and Technology, from June to September 2021.

**Results:**

The final symptom cluster scale for patients with MG consisted of 19 items(MGSC-19), with a content validity index ranging from 0.828 to 1.000 for each item and the content validity index was 0.980. Four common variables (ocular muscle weakness, general muscular weakness, treatment-related side effects, and psychiatric problems) were identified by exploratory factor analysis, which explained 70.187% of the total variance. The correlation coefficients between the scale dimension and the overall score ranged from 0.395 to 0.769 (all P < 0.01), while the correlation coefficients between dimensions varied from 0.324 to 0.510 (all P < 0.01). The Cronbach’s alpha, retest reliability, and half reliability were 0.932, 0.845, and 0.837, respectively.

**Conclusion:**

The validity and reliability of MGSC-19 were generally good. This scale can be employed to identify the symptom clusters to help healthcare givers develop individualized symptom management measures for patients with MG.

**Supplementary Information:**

The online version contains supplementary material available at 10.1186/s12883-023-03240-4.

## Introduction

Myasthenia gravis (MG) is a rare autoimmune disease of the neuromuscular junction, with the characteristic presentation of fatigable muscle weakness [1]. Patients with MG experience fluctuating symptoms that often require long-term relapse-preventive therapy [2]. The symptoms, such as ocular ptosis, diplopia, difficulty in making facial expressions and chewing, weakened limbs, and fatigue, have a negative impact on the quality of life (QOL) of patients and also impose an economic burden on their families and society [3–5]. In addition to traditional medical treatments, accurate and timely identification of symptoms and individualized management are crucial for caregivers to implement preventive health measures and improve the QOL of MG patients [6]. Currently, the assessment of symptoms using a symptom cluster scale appears to be a comprehensive and effective way to collect information.

However, previous studies on the symptoms of MG patients have mostly focused on the single-dimensional symptoms, without considering possible synergistic effects between symptoms. Although multi-symptom and multi-dimensional symptom assessment tools have been developed for cancer and other chronic diseases [7, 8], a specific, systemic, and reliable scale based on symptom clusters in MG is still lacking. Therefore, the aim of this study is to develop a multi-symptom, multi-dimensional symptom assessment scale for MG patients and to test its reliability. We hope this could pave the way for individualized symptom management by caregivers in the future.

## Methods

### Study design

**Fig. 1:**
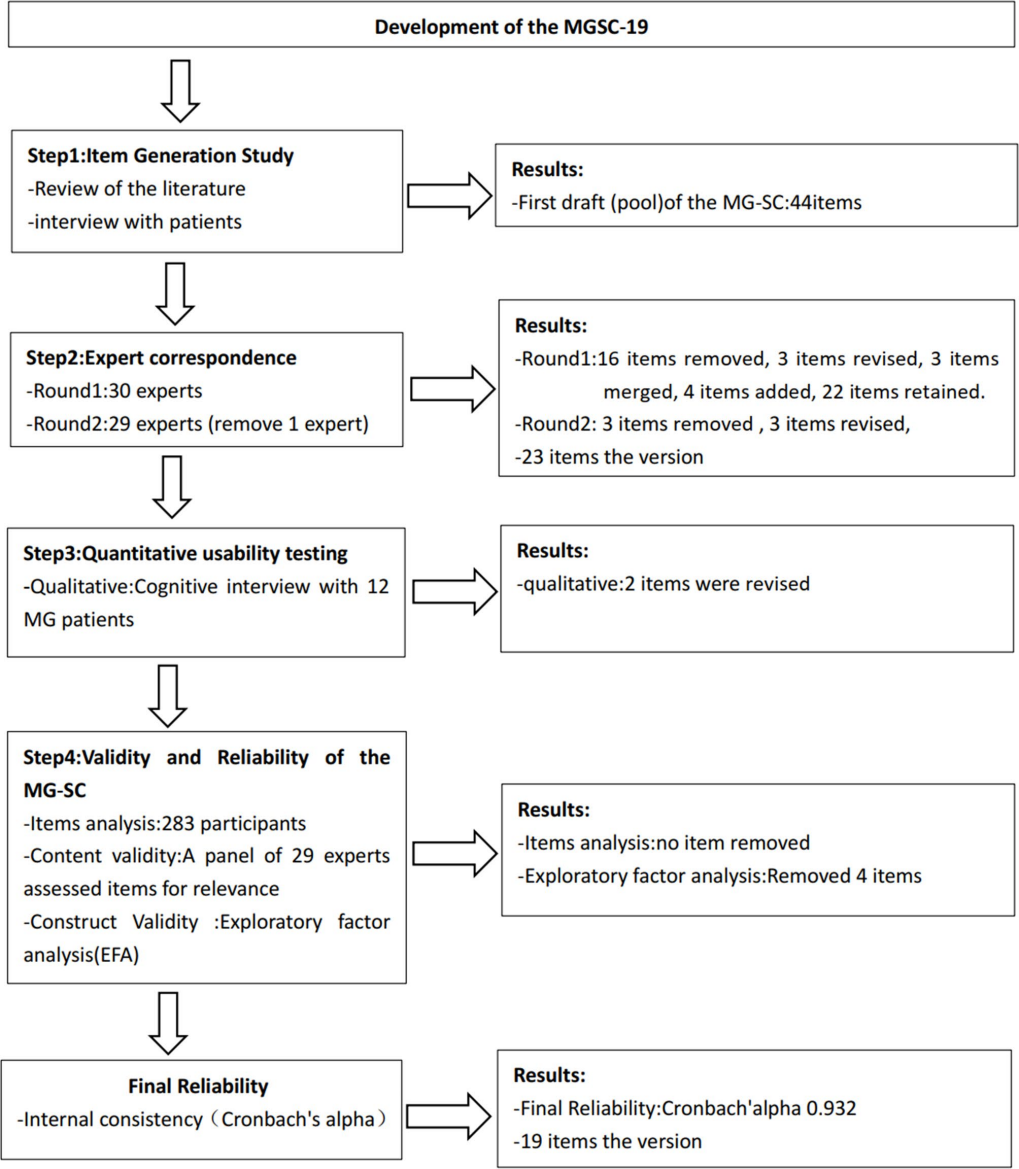
Process of developing and validating the MGSC-19.

The scale for symptom clusters in patients with MG(MGSC-19) was developed and verified in the following steps (Fig. 1): Steps 1, Item generation study; Steps 2: Expert correspondence. Steps 3: Quantitative usability testing. Steps 4: Validity and Reliability of the MG-SC. To verify the validity of the construct, exploratory factor analysis (EFA) was performed to determine factor structure.

### Instrument development

#### Theoretical foundations

The Theory of Unpleasant Symptoms (TOUS) [9] was used to guide the development of the scale draft. The TOUS expands the concept of ‘symptoms’ by their frequency, severity, distress and characteristics. As the ‘characteristics’ dimension is not appropriate for quantitative assessment, the scale has been developed to assess each symptom item on the remaining three dimensions (frequency, severity, and distress).

### Item generation

In order to build an item pool, we had referred to literature from the database established to March 2021 [10–14]. Based on the literature and the experiences from patients with MG, and the common symptoms of MG patients were summarized as a preliminary pool of symptom.

From April to June 2021, the qualitative interview was conducted in patients with MG. The sample size was based on the principle of data saturation, and the subjects were selected by the maximum variation method. Seventeen patients were finally included. Interview outline included: (1) The uncomfortable symptoms have you experienced so far since the first onset of the disease (2) What treatments (including medication and surgery) have you undergone since the onset of the disease, and What are the discomfort symptoms associated with these treatments? (3) How did you feel when faced with these uncomfortable symptoms? (4)Have these symptoms changed your lifestyle significantly? How do you like these changes? (5)What are your major concerns or feelings since your illness? Then, these data were analysed using the Colaizzi 7-step analysis method [15]. The results of the interviews were summarized and added to the initial pool of symptom entries, forming a final pool of 44 entries. All entries were scored positively on a 5-point Likert scale, with each symptom score being the sum of the frequency, severity and distress scores.

### Expert correspondence

Experts from general hospitals in 5 different provinces were invited to conduct 2 rounds of correspondence on the content of the scale. In the first round, 30 experts were included, and in the second round, as one expert who did not complete the questionnaire, 29 experts (including 15 medical experts; 10 clinical nursing experts; and 4 psychology experts) were included. The age of the experts (39.24 ± 4.75y), years of work (13.17 ± 7.56y), 19 with associate senior or higher positions, and 22 with master’s degrees or higher. 100% and 96.66% of the questionnaires were validly returned in the first and second round, respectively, and their authority coefficients of the experts were 0.880 and 0.888, with the Kendall harmony coefficients of the experts being 0.288 and 0.388, respectively (p < 0.001). In this study, items were selected based on the mean importance score of ≥ 4 and content validity index of ≥ 0.78. The entries were adjusted and modified based on the experts’ opinions. In the first round of consultation, 16 items were deleted, 3 items were revised, 3 items were merged, 4 items were added, and 22 items were retained. In the second round of consultation, 3 items were deleted and 3 items were combined, resulting in a test scale with 23 items.

### Cognitive interview

To avoid the ambiguity in understanding the questions, we cooperated 12 MG patients with the cognitive interviews on each item of the questionnaire [16]. The entry “insomnia” was changed into “sleep disorder”, and the entry “dysarthria” was replaced with “slurred speech” based on patients feedback.

### Reliability testing

#### Participants

A cross-sectional survey was conducted in patients with MG who were recruited from outpatient clinics at Tongji Hospital of Tongji Medical College, Huazhong University of Science and Technology, from June to September 2021. The inclusion criteria are as follows: (1) patients diagnosed with MG who met the diagnostic criteria of the Chinese guidelines for the diagnosis and treatment of MG (2020 version) [10]; (2) the disease course > 6 months; (3) age ≥ 18 years; (4) stable vital signs; (5) clear consciousness; (6) understanding the survey. Exclusion criteria: 1. Patients diagnosed as type V according to the clinical classification of Myasthenia gravis Foundation of America (MGFA) [17]; 2.Clinical diagnosis combined with other malignant tumors, mental diseases, or consciousness disorders; All subjects signed informed consent forms before the study. The questionnaire data was collected and managed by the investigator.

#### Item analysis

Item analysis is used to test the appropriateness of scales and their reliability. In this study, the following four methods were combined to assess the scale items: (1)Critical ratio method: The research participants were ranked based on their overall score. independent sample t-tests were done between the higher group(the top 27% of MG patients) and the lower one (last 27%). A critical ratio more than 3 and a p-value < 0.05 suggested that the entries had strong discrimination and should be kept. (2)Correlation coefficient method: If the correlation coefficient is 0.3 and P < 0.05, the item is very homogeneous with the overall scale. (3)Reliability test: If the Cronbach’s alpha value of the theoretical dimension to which an item belongs to increases considerably after deletion, it will be eliminated. (4)Commonality and factor loadings: Limit the number of retrieved components to one using principal component analysis. The Entry with commonality < 0.2 or factor loading < 0.45 will be removed [19]. SPSS 23.0 statistical software was used to analyse the data.

### Validity testing

Content validity: The importance of each item and its relation to the related content were evaluated by 29 experts, and the content validity index (CVI) of each item, dimension, and overall scale was generated based on the findings of the experts’ evaluation. Exploratory factor analysis, including principal component analysis and variance maximisation orthogonal rotation, was performed for structural validity. The test principles were as follows: (1) extracted factor eigenvalues > 1; (2) cumulative variance contribution of factors > 50%; (3) each factor contains at least two entries; (4) entries with loadings > 0.5 on one factor and low loadings on the other factors were removed; (5) entries with multiple loadings and close loadings (all loadings > 0.4 and difference 0.2) were removed; and (6) the gravel plot test principle was met and it was easy to name. The Spearman correlation coefficient was employed to assess the relationship between the components and the total. Criterion validity: the 15-item Myasthenia Gravis Quality of Survival Scale (MGQOL-15)has good reliability and validity [18], it is now widely used in clinical practice and was used as a validity standard to calculate the correlation between the scores and the scores of the scale developed for this study. Principal component analysis and maximum orthogonal rotation of variance were used to perform dimension reduction. For MGQOL-15 scale, a total of 3 common factors with Eigen values > 1 were extracted, with the cumulative variance contribution rate of 69.912%. Based on the loads and the content of items on dimensions, the 3 common factors can be named as follows: MG-related symptoms, Psychological conditions, and Mobility. These findings were consistent with a previous study by Wu H et al. (2018) [19].

### Reliability testing

Internal consistency reliability: Cronbach’s alpha coefficients and half-measure reliability were calculated for the total scale and each dimension. Retest reliability: Pearson’s correlation coefficient was used, and the number of retest samples was required to be at least 1/10 of the total study population. Thirty patients with MG were conveniently selected from the 283 formally measured patients, remeasured after 2 weeks and retest reliability was calculated.

### Optimal cut-off values

The optimal cut-off value of the MG patient symptom clusters scale to MG-QOL-15 was calculated using the receiver operating characteristic (ROC) curve analysis.

## Results

### Participant characteristics

In accordance with the requirement that the sample size should be at least 5–10 times the number of entries in the factor analysis, 295 questionnaires were distributed and 283 valid questionnaires were returned, with a valid return rate of 95.93%. Of the 283 patients, 95 (33.6%) were male and 188 (66.4%) were female; age ranged from 18 to 75 years; 125 (44.2%) were oculomotor patients and 158 (55.8%) were generalised MG; the duration of the disease ranged from 1 to 46 years; 195 (68.9%) of the patients had experienced a relapse of the disease. Of all the patients studied, 257 (83.7%) had taken or were taking cholinesterase inhibitors; 164 (58.0%) had taken or were taking glucocorticoids; and 149 (52.7%) had taken or were taking immunosuppressive drugs.

### Item analysis results

The results of the critical ratio method showed that the differences between the high and low groups were statistically significant (p < 0.001); the results of the correlation coefficient method showed that the correlation coefficients between the items and the total scale ranged from 0.395 to 0.769, indicating that the correlation between the items and the scale was within an acceptable range; the Cronbach’s alpha coefficient of the total scale was 0.944, and the removal of any one item in the scale did not increase the Cronbach’s alpha coefficient of the total scale. The Cronbach’s alpha coefficient for the total scale was 0.944, and the deletion of any one item in the scale did not increase the Cronbach’s alpha coefficient for the total scale, indicating that all items contributed to the internal consistency of the total scale. Using principal component analysis, the number of extracted factors was limited to 1, and the commonality was all > 0.2, so there was no need to delete entries. Based on the above four methods, no entries were removed from the initial scale.

### Results of the validity analysis

According to the results of the expert correspondence, the content validity index of the scale items ranged from 0.828 to 1.000, and the content validity index of the scale was 0.975. The 23 items were subjected to exploratory factor analysis, and the Kaiser-Meyer-Olkin(KMO) value was 0.932, which indicated excellent sampling adequacy and relatively compact patterns of correlation. Such factor analysis should produce distinct and reliable factors [20], and the Bartlett’s spherical test value was 4650.975(253 degrees of freedom, p < 0.001) which showed that it had an adequate relationship between the variables [21], indicating that the scale is suitable for factor analysis [16]. According to the aforementioned principles of structural validity, the scale was analysed using principal component analysis and maximum orthogonal rotation of variance, and the analysis was carried out by the “question-by-question deletion method” to obtain a valid interpreted scale structure. Four items (diarrhoea, nausea and vomiting, fatigue and shortness of breath) were deleted, and 19 items were retained. A total of four common factors with characteristic roots > 1 were extracted, and the cumulative variance contribution rate was 70.187%, as shown in Table 1. The gravel examination chart also indicates that it is appropriate to retain 4 common factors. Therefore, The final scale is divided into four dimensions: ocular muscle weakness, generalized muscle weakness, treatment-related side effects, and psychological disorders. The entry load of each dimension is 0.652–0.876 and are summarised in Table 2. Furthermore, although the dimensions were significantly intercorrelated in MGSC-19 (all p<0.01), these correlation coefficients were lower than those between the dimensions and total scores(Table 3), suggesting that the four dimensions were clustered rationally for MGSC-19. We also compared the MGSC-19 to MGQOL-15 (Table 4), which showed the two scales were significantly medium to high positive correlated in both dimensions and total scores, with the correlation coefficients of 0.270–0.703 (all p<0.01), indicating that MGSC-19 can effectively predict patients’ burdens.

**Table 1:** Exploratory factor analysis results of symptom assessment scale for patients with Myasthenia Gravis

Factors Analysis	KMO	Cumulative explained variation(%)	Delete entry					
Round 1	0.932	66.435	The factor loadings for “diarrhoea” were all < 0.5, deleted					
Round 2	0.932	67.887	The factor loadings for “nausea and vomiting” were all < 0.5, deleted					
Round 3	0.930	69.361	“Fatigue” factor 1, factor 2 and factor 3 loadings are all > 0.4 and the difference is < 0.2, deleted					
Round 4	0.924	69.839	“Shortness of breath” factor 1 and factor 2 loadings are > 0.4 and the difference is < 0.2, deleted					
Round 5	0.921	70.187						

**Abbreviation**: KMO = Kaiser-Meyer-Olkin.

**Table 2:** Factors, items and factor loadings of The MG-SC (N = 283)

Symptom clusters	symptom	Factor loadings			
		1	2	3	4
Ocular muscle weakness	ptosis	0.148	0.139	0.102	**0.828**
	diplopia	0.228	0.177	0.179	**0.741**
Generalized muscle weakness	masticatory atonia	**0.819**	0.105	0.092	0.239
	slurred speech	**0.764**	0.133	0.030	0.264
	dysphagia	**0.876**	0.115	0.125	0.158
	sensation of foreign body in pharynx	**0.794**	0.149	0.061	0.163
	hoarseness	**0.729**	0.206	-0.007	0.153
	head up in difficulty	**0.793**	0.191	0.152	0.109
	Upper limb weakness	**0.758**	0.213	0.164	0.059
	Lower limb weakness	**0.730**	0.230	0.223	0.012
	dyspnea	**0.787**	0.177	0.240	0.024
	weak cough	**0.802**	0.222	0.181	0.056
	chest tightness	**0.660**	0.396	0.305	-0.076
Treatment-related side effects	Weight change	0.247	0.223	**0.810**	0.125
	moon-face	0.190	0.128	**0.854**	0.185
Psychological disorders	anxiety	0.243	**0.814**	0.142	0.141
	depression	0.172	**0.864**	0.076	0.114
	sleep disorder	0.246	**0.652**	0.193	0.008
	stigma	0.171	**0.748**	0.048	0.182
Eigenvalue		6.967	2.988	1.804	1.577
Percentage of variance		36.668	15.725	9.494	8.3

**Abbreviation**: MG-SC = The Symptom Cluster scale for patients with myasthenia gravis

**Table 3:** Correlation coefficients between dimensions and correlation coefficients between dimensions and total table (r value)

Item	Ocular symptom clusters	General symptom clusters	Treatment-related side effects	Psychological disorders
Ocular symptom clusters	1			
General symptom clusters	0.329**	1		
Treatment-related side effects	0.324**	0.427**	1	
Psychological disorders	0.334**	0.510**	0.400**	1
MGSC-19 sum	0.539**	0.909**	0.625**	0.716**

** Significant correlation at 0.01 level (two-tailed)

**Abbreviation**: MGSC-19: 19-Item the Symptom Cluster scale for patients with MG.

**Table 4:** Correlation between the scale for symptom clusters in patients with myasthenia gravis (MGSC-19) and the 15-Item Quality of Life Instrument for myasthenia gravis scale (MGQOL-15) (r value)

Item	MG-related symptoms	Psychological conditions	Mobility	Total scores of MGQOL-15
Ocular symptom clusters	0.435**	0.362**	0.382**	0.418**
General symptom clusters	0.703**	0.413**	0.620**	0.640**
Treatment-related side effects	0.320**	0.270**	0.396**	0.385**
Psychological disorders	0.492**	0.688**	0.587**	0.656**
Total scores of MGSC-19	0.722**	0.558**	0.702**	0.736**

** Significant correlation at 0.01 level (two-tailed)

### Reliability testing results

The Cronbach’s alpha coefficient for the total scale was 0.932 and the Cronbach’s alpha coefficients for the dimensions were 0.950, 0.829, 0.775 and 0.632 respectively. The Cronbach’s alpha coefficients were 0.950, 0.829, 0.775 and 0.632 for each dimension respectively. The half-measure reliability of the total scale was 0.837 and the half-measure reliability of each dimension was 0.888, 0.831, 0.775 and 0.632 respectively. The retest reliability for the total scale was 0.845 and the retest reliability for each dimension was 0.724, 0.876, 0.781 and 0.870 respectively.

### Optimal cut-off values

**Fig. 2:**
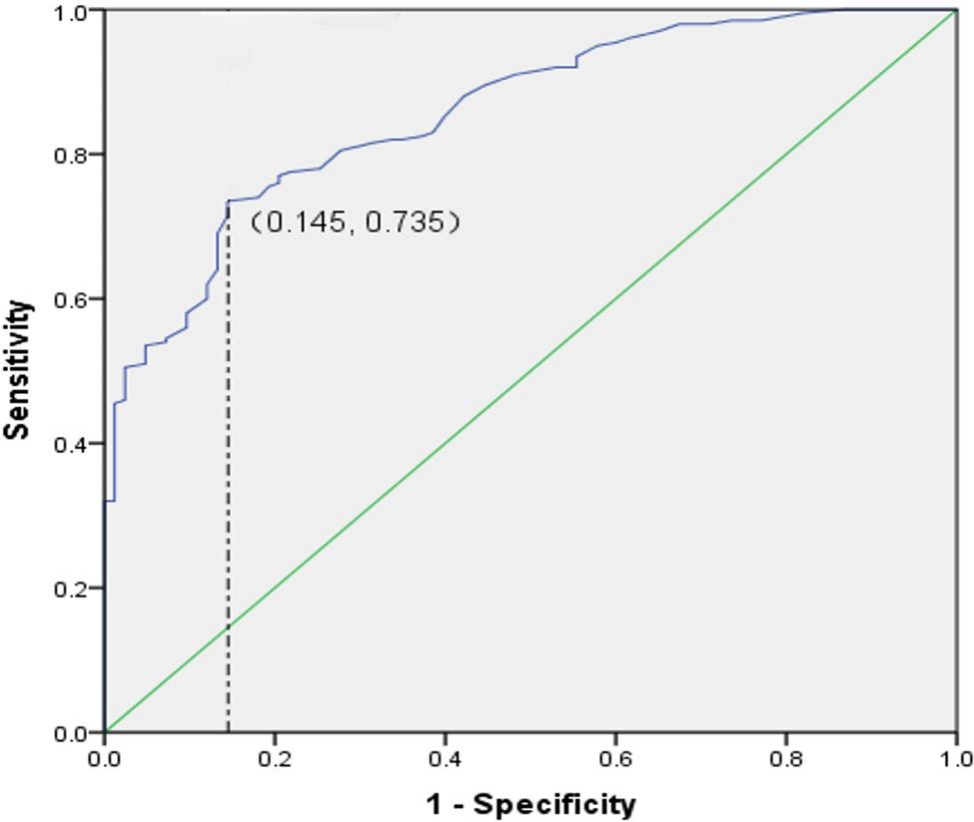
The ROC curve of MGSC-19 for predicting quality of life The optimal cut-off value for the MGSC-19 scale was 102, with a sensitivity of 73.5% and specificity of 85.5%

Based on previous research [22], we defined a score of < 12 on the MGQOL-15 as indicating mild impact on daily life, and a score of ≥ 12 as indicating severe impact. We then plotted ROC curves (Fig. 2) and determined that the optimal cut-off value for the MGSC-19 scale was 102, with a sensitivity of 73.5% and specificity of 85.5% .

## Discussion

Guided by unpleasant symptom theory, the present study attempted to develop reliable measurement tool to identify the symptom clusters of patients undergoing MG. The symptom assessment scale for MG was divided into three dimensions: frequency, severity and distress, which enriched the meaning of “symptoms”, which can be used to accurately and comprehensively assess a patient’s symptoms and provide a basis for targeted symptom management by clinical staff.

The scale was created by a rigorous procedure. To begin, a thorough literature review of domestic and foreign research on the symptoms of MG patients was undertaken to create a preliminary pool of items, which was then complemented with the findings of qualitative interviews. The 29 specialists surveyed were from five provinces and included medical professionals, clinical nursing experts, and psychologists who were geographically represented and authoritative in their fields. Following the development of the scale, 12 patients were chosen for cognitive interviews, assuring a high degree of readability. A high sample size was used in this study throughout the scale’s reliability testing phase to confirm the data’s legitimacy. Therefore, the scale can accurately assess the symptoms of MG patients and has a high validity.

The validity scales’ content validity, structural validity, and correlational validity are all part of their validity. The content validity index of each item in this study varied from 0.828 to 1.000, and the entire scale’s content validity index was 0.975, suggesting that the scale’s content and distribution were appropriate. The starting item pool was expanded from 44 to 19, in accordance with the requirement that the first item pool be at least 50% greater than the pool of items for the entire scale. During the entrance analysis, four approaches were applied for entry screening, confirming the scale’s homogeneity. Exploratory factor analysis was used to identify the best probable structure of the scale, and the “question-by-question deletion procedure” was used to minimise the number of entries. Finally, 19 items were maintained, and the common components with eigenvalues greater than one were retrieved as the scale’s four dimensions, explaining 70.187% of the variance collectively. Factor 1 is a collection of 11 general symptoms (weakness in chewing, slurred speech, trouble swallowing, foreign body sensation in the throat, hoarseness, difficulty elevating the head, weakness in upper limbs, weakness in lower limbs, difficulty breathing, weakness in coughing, chest tightness). Factor 2 is the group of psychological symptoms, which includes four items (anxiety, sadness, sleep disruption, and shame); Factor 3 is the group of pharmacological side effects, which includes two items (weight gain and weight loss). Factor 4 is the ocular symptom cluster, which has two elements (ptosis, diplopia). Furthermore, although the dimensions were significantly intercorrelated in MGSC-19, these correlation coefficients were lower than those between the dimensions and total scores, suggesting that the four dimensions were clustered rationally for MGSC-19. We also compared the MGSC-19 to MGQOL-15, which showed the two scales were significantly correlated in both dimensions and total scores, indicating that MGSC-19 can effectively predict patients’ burdens. The Cronbach’s alpha coefficient for the total scale in this study was 0.932, the folded half reliability was 0.837, and the Cronbach’s alpha coefficient and folded half reliability for each dimension were greater than 0.6. The retest reliability of the scale after 2 weeks was 0.845, indicating that the scale has good stability.

Rapid identification of disease-related symptoms is a weak point in the self-management process for patients with MG [23]. Due to the low prevalence of MG, as well as the fact that some symptoms, such as diplopia, are very obvious to patients while others, such as lower limb weakness, are not obvious in the early stages, patients frequently attribute this to overwork or lack of exercise and may not believe it is related to the disease. The present stage of symptom evaluation instruments for people with MG is insufficient to measure patients’ symptoms. Mullins et al. [24]summarized the symptoms of patients with MG in their results involving Mobility, Symptoms, General Contentment, Emotional Well-being. The most widely used 15-Item Quality of Life Instrument for myasthenia gravis scale (MG-QOL15) [18]covers the physical, social and psychological domains, but does not take into account the treatment-related symptoms of the patient. In fact, for MG patients, long-term medication is usually required to control symptoms. These therapies may have adverse effects in addition to symptom alleviation and may interact synergistically with the symptoms, thereby impacting the patient’s quality of life [25]. This study identifies MG symptom clusters through exploratory factor analysis with concise symptom entries, allowing patients to recognise and control their symptoms more quickly. Furthermore, the current study compiled the symptom assessment scale for MG patients in a scientific and rigorous process, focusing on patients’ subjective feelings, representing the most comprehensive content with the fewest entries, with good sensitivity and specificity, and with strong practicality and clinical appropriateness, which can help patients correctly recognise their symptoms, facilitate their self-monitoring of disease progression, and provide a foundation for future research.

### Limitations

In this study, only exploratory factor analysis was used to evaluate the structural validity of the scale, and no additional validation factor analysis was performed to confirm the scale’s scientific validity and appropriateness. Furthermore, the study’s sample was obtained from one medical centre, and more patients from multiple centers are needed to validate our results.

## Conclusions

In conclusion, physiologic testing from this study indicated that the symptom cluster assessment scale for MG patients is valid and reliable. We suggest that this assessment tool can be employed to identify the symptom clusters of patients with MG in the clinical setting. This type of identification helps healthcare practitioners to deliver effective therapies to relieve patients’ symptom suffering.

## Electronic supplementary material

Below is the link to the electronic supplementary material.

Supplementary Material 1

## Data Availability

The datasets used and/or analyzed during the current study are available from the corresponding author on reasonable request.

## Abbreviations

MG: Myasthenia gravis
QOL: Quality of life
TOUS: The unpleasant symptom theory
MGSC-19: 19-Item the Symptom Cluster scale for patients with MG
EFA: Exploratory factor analysis
MGFA: Myasthenia gravis Foundation of America
CVI: The content validity index
MGQOL-15: 15-Item Quality of Life Instrument for myasthenia gravis scale
