# Supplementary material for: Development and validation of the scale for symptom clusters in patients with myasthenia gravis
Source: BMC Neurol. 2023 May 19;23:196. doi: 10.1186/s12883-023-03240-4 (PMC10197207; doi:10.1186/s12883-023-03240-4)
Supplement: Supplementary file 1 — Supplementary Material 1 [file 12883_2023_3240_MOESM1_ESM.docx]

**Supplementary table1. Symptom Clusters Scale for Patients with MG**

| Symptom clusters | Number | Item | Frequency | | | | | Severity | | | | | Degree of distress | | | | |
| --- | --- | --- | --- | --- | --- | --- | --- | --- | --- | --- | --- | --- | --- | --- | --- | --- | --- |
|  |  |  | Never | A little bit | Somewhat | Quite a lot | Extremely | Never | A little bit | Somewhat | Quite a lot | Extremely | Never | A little bit | Somewhat | Quite a lot | Extremely |
| ocular muscle weakness | 1 | ptosis |  |  |  |  |  |  |  |  |  |  |  |  |  |  |  |
|  | 2 | diplopia |  |  |  |  |  |  |  |  |  |  |  |  |  |  |  |
| generalized muscle  weakness | 3 | masticatory atonia |  |  |  |  |  |  |  |  |  |  |  |  |  |  |  |
|  | 4 | slurred speech |  |  |  |  |  |  |  |  |  |  |  |  |  |  |  |
|  | 5 | dysphagia |  |  |  |  |  |  |  |  |  |  |  |  |  |  |  |
|  | 6 | sensation of foreign body in pharynx |  |  |  |  |  |  |  |  |  |  |  |  |  |  |  |
|  | 7 | hoarseness |  |  |  |  |  |  |  |  |  |  |  |  |  |  |  |
|  | 8 | head up in difficulty |  |  |  |  |  |  |  |  |  |  |  |  |  |  |  |
|  | 9 | Upper limb weakness |  |  |  |  |  |  |  |  |  |  |  |  |  |  |  |
|  | 10 | Lower limb weakness |  |  |  |  |  |  |  |  |  |  |  |  |  |  |  |
|  | 11 | dyspnea |  |  |  |  |  |  |  |  |  |  |  |  |  |  |  |
|  | 12 | weak cough |  |  |  |  |  |  |  |  |  |  |  |  |  |  |  |
|  | 13 | chest tightness |  |  |  |  |  |  |  |  |  |  |  |  |  |  |  |
| treatment-related side effects | 14 | Weight change |  |  |  |  |  |  |  |  |  |  |  |  |  |  |  |
|  | 15 | moon-face |  |  |  |  |  |  |  |  |  |  |  |  |  |  |  |
| psychological disorders | 16 | anxiety |  |  |  |  |  |  |  |  |  |  |  |  |  |  |  |
|  | 17 | depression |  |  |  |  |  |  |  |  |  |  |  |  |  |  |  |
|  | 18 | sleep disorder |  |  |  |  |  |  |  |  |  |  |  |  |  |  |  |
|  | 19 | stigma |  |  |  |  |  |  |  |  |  |  |  |  |  |  |  |
